# The Impact of the COVID-19 Pandemic and Emergency Distance Teaching on the Psychological Status of University Teachers: A Cross-Sectional Study in Jordan

**DOI:** 10.4269/ajtmh.20-0877

**Published:** 2020-10-27

**Authors:** Amal Akour, Ala’a B. Al-Tammemi, Muna Barakat, Rama Kanj, Hussam N. Fakhouri, Ahmad Malkawi, Ghadeer Musleh

**Affiliations:** 1 Department of Pharmacy, Faculty of Pharmacy, Al-Zaytoonah University of Jordan, Amman, Jordan;; 2 Department of Biopharmaceutics and Clinical Pharmacy, School of Pharmacy, The University of Jordan, Amman, Jordan;; 3 Department of Epidemiology and Global Health, Faculty of Medicine, Umeå University, Umeå, Sweden;; 4 Doctoral School of Health Sciences, University of Debrecen, Debrecen, Hungary;; 5 Department of Clinical Pharmacy and Therapeutics, Faculty of Pharmacy, Applied Science Private University, Amman, Jordan;; 6 School of Psychology and Clinical Language Sciences, University of Reading, Reading, United Kingdom;; 7 Department of Computer Science, King Abdullah II School for Information Technology, The University of Jordan, Amman, Jordan;; 8 Department of Health Promotion, School of Nutrition and Translational Research in Metabolism (NUTRIM), Maastricht University Medical Center, Maastricht University, Maastricht, The Netherlands;; 9 Department of Family and Community Medicine, School of Medicine, The University of Jordan, Amman, Jordan

## Abstract

The COVID-19 pandemic has struck many countries globally. Jordan has implemented strict nationwide control measures to halt the viral spread, one of which was the closure of universities and shifting to remote teaching. The impact of this pandemic could extend beyond the risk of physical harm to substantial psychological consequences. Our study aimed at assessing 1) psychological status, 2) challenges of distance teaching, and 3) coping activities and pandemic-related concerns among university teachers in Jordan in the midst of COVID-19–related quarantine and control measures. We conducted a cross-sectional study using an anonymous online survey. The measure of psychological distress was obtained using a validated Arabic version of the Kessler Distress Scale (K10). Other information collected included sociodemographic profile, methods used to handle distress, motivation to participate in distance teaching, and challenges of distance teaching as well as the most worrisome issues during this pandemic. Three hundred eighty-two university teachers returned completed surveys. Results of K10 showed that 31.4% of respondents had severe distress and 38.2% had mild to moderate distress. Whereas gender was not associated with distress severity, age had a weak negative correlation (Rho = −0.19, *P* < 0.0001). Interestingly, most teachers had moderate to high motivation for distance teaching. Engagement with family was the most reported self-coping activity. More than half of the participants were most concerned and fearful about SARS-CoV-2 infection. In conclusion, university teachers have shown to exhibit various levels of psychological distress and challenges during the implementation of precautionary national measures in the battle against COVID-19 in Jordan.

## INTRODUCTION

Recently, the WHO avowed a pandemic infectious disease caused by a novel coronavirus, namely, SARS-CoV-2, and has been referred to as COVID-19.^1^ COVID-19 has been reported as an extraordinary and very contagious disease.^2–4^ SARS-CoV-2 infection leads to a variety of respiratory symptoms which may range from mild to severe degrees and might end up with fatal consequences leading to death.

Since December 2019, COVID-19 has rapidly transmitted from China to many countries worldwide as a result of international travel and became one of the global challenges that posed a significant burden on the health sector as well as other sectors considering the current lack of a vaccine or any specified antiviral therapy. As of July 17, 2020, the number of confirmed cases and deaths over the world was 13,616,593 and 585,727, respectively. Besides, 216 countries, areas, or territories have been struck by the disease.^5^ Consequently, decision-makers of various sectors declared strict confinement measures to control this global pandemic at national and international levels.^6^

In the middle of March 2020, part of the Jordanian national response to halt the spread of COVID-19 included critical decisions that imposed a general public quarantine and a closure of all the educational institutions, including schools, colleges, and universities. As a consequence of that closure, an emergency remote learning and teaching strategy (distance learning/teaching) was officially used, assisting the completion of the already started academic courses at various higher academic institutions in the country.^7,8^ As of July 17, 2020, 1,209 confirmed cases, 1,021 recovered cases, and 10 deaths were attributed to COVID-19 in Jordan.^8^

In addition to the biological and physical harm related to COVID-19, psychological impacts are highly expected too. The mental health impacts of the COVID-19 pandemic are not age or gender related, as these impacts could affect individuals irrespective of their sociodemographic differences.^9–11^ Ho et al.^11^ reported that any individual could suffer from new psychiatric symptoms, even without having a previous history of a psychiatric illness. Symptoms such as anxiety, fear of infection and death, anger, hopelessness, stigma, and blame all could happen during such pandemic.^12^ On a more advanced level, the previously mentioned psychological symptoms may evolve into a well-established psychiatric illness such as post-traumatic stress symptomology, depression, paranoia, panic, delirium, and suicidal ideation, especially among youngers who have high self-blame.^11–13^

Concerning the higher education sector, university students are believed to be one of the vulnerable groups who could be afflicted by various degrees of psychological distress during this pandemic, and this has been reported in some studies.^14,15^ University teachers (academic staff) at higher education institutions (HEIs) as well could be impacted psychologically by the consequences of the current pandemic and its precautionary mitigation rules including online remote teaching. In Jordan, and before the COVID-19 crisis, a few number of courses at various universities had been delivered to students remotely in the form of asynchronous online lectures; however, the vast majority of courses require in-person attendance to classrooms for both the students and teachers.^16,17^ In addition, a national strategy (2007–2010) proposed by the Jordanian Ministry of Higher Education (MoHE) had addressed the need for appropriate and effective integration of online remote learning tools with on-campus teaching activities, but the transformation process is still considered a “challenging pedagogy” of the learning process in Jordan, where the accreditation of online degrees is very strict. Despite the widespread high-speed Internet offered by various Internet service providers (ISPs) in the country,^16–19^ universal access to high-quality Internet in Jordan is not always guaranteed. Since the suspension of on-campus academic activities because of the current pandemic, the MoHE along with Ministry of Digital Economy and Entreprenuership, Jordanian universities, and ISPs have taken more serious steps to implement online remote learning and teaching coupled with numerous strategies to ensure a quality higher education.^20^

## STUDY AIMS

In Jordan, declaring the suspension of face-to-face on-campus academic activities at HEIs and shifting to emergency distance learning and teaching since the middle of March 2020 were among the nationally declared precautionary and control measures to fight COVID-19. The latter strategy imposed an unprecedented academic experience on both students as well as the academic staff, which can lead to unexpected outcomes.

Therefore, our study aims were 1) to assess the psychological well-being of university teachers at various HEIs in Jordan in the midst of COVID-19–related quarantine and control measures; 2) to explore the challenges of the emergency remote teaching strategy among academicians and any associated technostress, that is, the stress related to using technologies and their effects on psychological status^21^; and 3) to explore different self-coping activities and any pandemic-related concerns among the university teachers.

## METHODS AND INSTRUMENTS

### Study design and participants.

We conducted a cross-sectional study in the period May 14–27, 2020, using an anonymous web-based survey. A snowball convenience sampling strategy was used to recruit participants (university teachers) through social media, that is, Facebook, WhatsApp groups of academic staff, and LinkedIn, in addition to institutional emails. The university teachers who had an interest to participate could open a link and receive a detailed cover letter with electronic informed consent. The participants did not receive any form of compensation on participation in this study.

Because of the unfolding situation of COVID-19, data collection was carried out using an online survey because of the rules of physical distancing coupled with the closure of on-campus teaching activities at all HEIs in Jordan at the time of conducting the study. In addition, using an online survey helped to eliminate the geographical boundaries, thus reaching potential participants from different cities and regions in Jordan, and this has been addressed in a recent systematic review that reported the effective use of social media platforms in health and psychological research studies.^22^ It was estimated that faculty members at HEIs in Jordan constitute around 11,000 academics of different ranks^23^; hence, by using Open Source Epidemiologic Statistics for Public Health (OpenEpi, Atlanta, GA) software version 3.01, a sample size of at least 372 participants was required for our study (with 95% confidence level and 5% margin of error).

Several eligibility criteria for participation were implemented, including 1) resident in Jordan during the pandemic, 2) actively appointed as a university teacher (lecturer, assistant professor, associate professor, and professor) at one of the HEIs in Jordan, and 3) consented to participate in this study voluntarily.

### Survey instrument and related measures.

Google Form^®^ (Google LLC, Mountain View, CA) was used to create the online survey which was designed in modern standard Arabic. Three sections with overall 29 questions were included in this anonymous online survey. The first section consisted of 13 questions distributed as follows: 11 questions about participants’ sociodemographic profile including age, gender, marital status, smoking status, the region of residence, region of the HEI, type of the HEI (public versus private), scientific discipline, academic ranking, and the duration of teaching experience. Also, participants’ previous psychiatric history and related medication usage were explored by two questions included in this section.

In the second section of the online survey, we used an Arabic version of the Kessler Distress Scale (K10). The Arabic version of K10 is provided by Harvard Medical School.^24^ K10 is one of the internationally validated tools for rapid assessment of psychological distress and includes 10 questions with responses in the form of five-point intensity Likert scale.^25–28^ In a recent study, the Arabic version of K10 has shown satisfactory psychometric properties and reliability with a Cronbach’s α equals 0.88.^28^

K10 questionnaire comprised a series of 10 questions about the self-reported feelings in last 30 days: “feeling tired out for no good reason,” “feeling nervous,” “feeling so nervous that nothing could calm you down,” “feeling hopeless,” “feeling restless or fidgety,” “feeling so restless you could not sit still,” “feeling depressed,” “feeling that everything was an effort,” “feeling so sad that nothing could cheer you up,” and “feeling worthless.”^27^

The five-point intensity Likert-scale responses to K10 questions are as follows: “1 = none of the time,” “2 = a little of the time,” “3 = some of the time,” “4 = most of the time,” and “5 = all of the time,” Thus, the overall K10 score ranges between 10 and 50. Besides, if a respondent answered questions two and five of K10 as “none of the time,” then questions three and six were scored as one point according to K10 scoring guide.^27^

Concerning severity of distress, the following categories, that is, no distress, mild distress, moderate distress, and severe distress, were applied according to the overall K10 score for each category: (10–19), (20–24), (25–29), and (30–50), respectively.^27^

The third section of this online survey included six questions about 1) self-adaptive activities 2) whether participants have used medicinal drugs because of pandemic-induced distress or not, 3) university teachers’ motivation for remote teaching, 4) perceived challenges of distance teaching among teachers, 5) commonly used distance teaching platforms, and last, 6) major pandemic-related concerns as perceived by the teachers.

For the purpose of assessing face validity, phrasing, and clarity of this survey, Amal Akour approached 15 university teachers and researchers from various academic disciplines who were asked to join a piloting phase. This piloting phase resulted in a minor modification of the initial version of the survey (linguistic modification). The responses from the piloting phase were not included in our analysis.

### Data analyses.

Data were extracted from completed surveys and then incorporated into STATA IC 16.1 (StataCorp LLC., College Station, TX) for analysis. Continuous variables were presented using summary and descriptive statistics, whereas categorical variables were presented as frequencies and percentages. As our sampling strategy was a non-probabilistic (non-randomized), thus, some of the nonparametric tests were used. To assess the correlation between overall K10 scores and different genders, we used the Wilcoxon rank-sum test, whereas Spearman’s rank correlation was used to assess the relationship between overall K10 score and age.

Multiple logistic regression analysis was used to look for any association between distress severity levels and other independent predictors such as age, gender, marital status, scientific discipline, motivation for distance teaching, duration of teaching experience, academic rank, and previous history of psychiatric illness. The psychological distress severity levels were dichotomized and recoded to achieve a binary outcome-dependent variable ([0 = no to mild distress] and [1 = moderate to severe distress]).

The same analysis was used to test the association between motivation for distance teaching (outcome-dependent variable) and other predictors including age, gender, scientific discipline, academic rank, and duration of teaching experience. The motivation for distance teaching was further recoded into a binary outcome variable ([0 = no to low motivation] and [1 = moderate to high Motivation]) to meet the requirement of multiple logistic regression analysis. The confidence level was set at 95%. A *P*-value < 0.05 was considered statistically significant.

### Ethical considerations.

This study was granted an exemption from institutional review board (IRB) review by the head of the IRB committee at Al-Zaytoonah University of Jordan dated May 13, 2020, considering the survey and the overall study carry no risk to participants. This study was conducted conforming to the Declaration of Helsinki and the code of conduct of research on human subjects in the country. The survey ensured the confidentiality and anonymity of the study participants. Moreover, a cover letter was included in the survey describing the nature and objectives of our study, inclusion criteria for participation, voluntary participation, and withdrawal. Furthermore, interested participants were requested to provide informed consent (electronic).

## RESULTS

### Sociodemographic profile of participants.

Three hundred eighty-seven surveys were received. Five surveys were incomplete because the respondents did not consent to participate in the study, whereas the remaining who completed 382 surveys were included in the analysis. The number of male participants was higher than that of females (*n* = 212, 55.5% and *n* = 170, 44.5%; respectively). The mean age was 43.9 years (SD = 9.9) and ranged between 25 and 75 years, with predominantly Jordanian nationality (*n* = 354, 92.7%). The duration of teaching experience ranged between 1 and 53 years, with a mean of 12.2 years (SD = 9.5). The majority of participants were married (*n* = 292, 76.4%), currently nonsmokers (*n* = 266, 69.6%), residing in central Jordan (*n* = 262, 68.6%), working at public HEIs (*n* = 265, 69.4%), with academic rank as assistant professor (*n* = 150, 39.3%), from medical discipline (*n* = 156, 40.8%), and with no previous psychiatric history (*n* = 368, 96.3%). However, only 14 participants (3.7%) were with a history of a psychiatric condition, among whom five participants reported the current use of psychiatric medications for their preexisting conditions. The detailed sociodemographic profile of the respondents is provided in Table 1.

**Table 1:** Sociodemographic profile of participants

Number of respondents	382
Gender, *n* (%)	
Male	212 (55.5)
Female	170 (44.5)
Age-group (mean, SD, range) (years), *n* (%)	43.9, 9.9, 25–75
25–34	63 (16.5)
35–44	153 (40.0)
45–54	105 (27.5)
55–64	50 (13.1)
≥ 65	11 (2.9)
Nationality, *n* (%)	
Jordanian	354 (92.7)
Non-Jordanian	28 (7.3)
Marital status, *n* (%)	
Single	74 (19.4)
Married	292 (76.4)
Divorced/separated/widowed	16 (4.2)
Smoking status, *n* (%)	
Smoker	116 (30.4)
Currently nonsmoker	266 (69.6)
Region of residence, *n* (%)	
Northern governorates	76 (19.9)
Central governorates	262 (68.6)
Southern governorates	44 (11.5)
Academic institution, *n* (%)	
Public university/college	265 (69.4)
Private university/college	117 (30.6)
Academic rank, *n* (%)	
Lecturer	70 (18.3)
Assistant professor	150 (39.3)
Associate professor	86 (22.5)
Full professor	76 (19.9)
Duration of teaching experience (mean, SD, range)	12.2, 9.5, 1–53
Scientific discipline, *n* (%)	
Medical/health	156 (40.8)
Humanities	113 (29.6)
Sciences	113 (29.6)
Region of the academic institution, *n* (%)	
Northern Jordan	82 (21.5)
Central Jordan	240 (62.8)
Southern Jordan	60 (15.7)
History of preexisting psychologic/psychiatric condition, *n* (%)	
Yes	14 (3.7)
No	368 (96.3)
Current psychiatric medication usage among participants (*n* = 14)	
Yes	*n* = 5
No	*n* = 9

### Psychological distress results.

**Figure 1.**
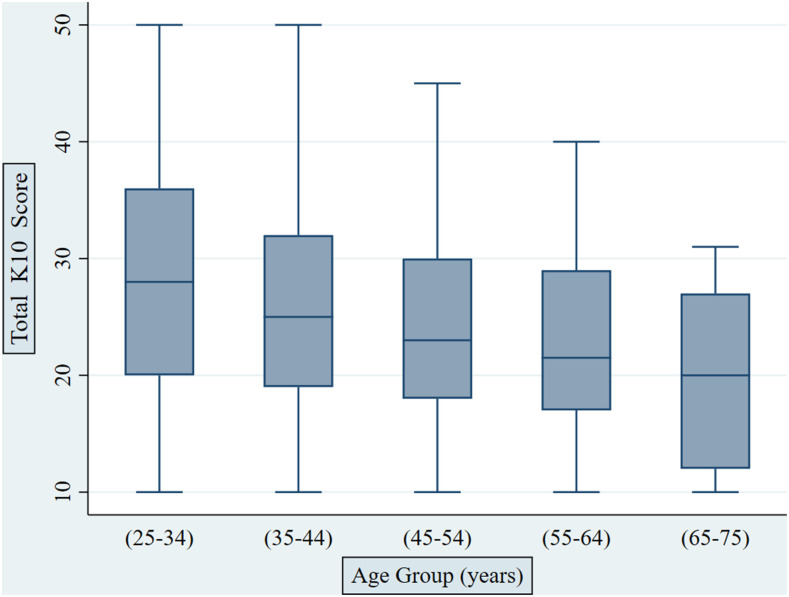
Overall (total) Kessler Distress Scale (K10) scores among different age-groups of participants. This figure appears in color at www.ajtmh.org.

The overall K10 scores had a mean of 25.6 (SD = 8.7), with a minimum and maximum score of 10 and 50, respectively. The most encountered categories, as defined by K10 categorization, were severe distress (*n* = 120, 31.4%) followed by no distress (*n* = 116, 30.4%). In addition, the overall K10 score was not significantly correlated with gender as found by the Wilcoxon rank-sum test, that is, males (mean K10 score = 24.7, SD = 8.7) versus females (mean K10 score = 25.5, SD = 8.7), with *P* = 0.38. On the other hand, age had a statistically significant, albeit weak, inverse relationship with the overall K10 score as found by Spearman’s rank correlation (Rho = −0.19, *P* < 0.0001), which indicates that the younger the age, the more likely to possess the high overall K10 score, thus more psychological distress (Figure 1).

Among the 14 participants who reported a previous history of psychological conditions, only nine of them were within severe psychological distress category, whereas three participants were within no psychological distress category. The remaining two participants were distributed among mild and moderate psychological distress categories, one for each. The multiple logistic regression analysis has revealed a statistically significant association between psychological distress severity and age categories, that is, with an increase of age, there is less likelihood of having a moderate to severe distress, which supports the Spearman’s rank correlation finding. Besides, a statistically significant association was found between low motivation for distance teaching and distress severity (adjusted odd ratio [aOR] = 2.42, *P* = 0.006, 95% CI: 1.28–4.55), which indicates that university teachers who had low motivation for distance teaching are more likely to have 1.42 more odds of suffering from moderate to severe distress than the reference category. The detailed results of multiple logistic regression analysis are demonstrated in Table 2. Moreover, descriptive details about the psychological distress severity levels among respondents are provided in Table 3.

**Table 2:** Results of multiple logistic regression for the association between psychological distress severity (recoded as binary outcome) and multiple predictor variables

Predictor	Crude OR (95% CI)	*P*-value	Adjusted OR (95% CI)	*P*-value
Age-group (years)				
25–34	Reference			
35–44	0.58 (0.32–1.06)	0.079	0.55 (0.27–1.11)	0.096
45–54	0.50 (0.27–0.95)	0.036*	0.38 (0.16–0.86)	0.020*
55.64	0.32 (0.15–0.70)	0.004*	0.18 (0.06–0.53)	0.002*
65–75	0.22 (0.052–0.89)	0.035*	0.10 (0.02–0.60)	0.012*
Gender				
Female	Reference			
Male	0.85 (0.57–1.28)	0.436	0.94 (0.59–1.50)	0.809
Marital status				
Divorced	Reference			
Married	0.76 (0.25–2.30)	0.625	1.22 (0.37–4.04)	0.743
Single	1.06 (0.33–3.48)	0.917	1.08 (0.31–3.81)	0.900
Widowed	1.71 (0.12–23.94)	0.689	3.43 (0.21–56.33)	0.387
Psychiatric illness				
No	Reference			
Yes	2.70 (0.83–8.76)	0.099	2.50 (0.74–8.43)	0.142
Duration of experience (years)				
1–15	Reference			
16–35	1.05 (0.64–1.73)	0.854	1.82 (0.92–3.63)	0.088
36–53	0.61 (0.26–1.45)	0.264	1.65 (0.50–5.45)	0.415
Academic rank				
Assistant professor	Reference			
Associate professor	1.12 (0.66–1.91)	0.674	1.42 (0.78–2.56)	0.248
Full professor	0.95 (0.55–1.66)	0.857	1.37 (0.65–2.86)	0.409
Lecturer (MSc)	1.76 (0.99–3.13)	0.054	1.38 (0.73–2.63)	0.326
Scientific discipline				
Humanities	Reference			
Medical	1.02 (0.63–1.65)	0.943	0.88 (0.52–1.49)	0.633
Sciences	0.90 (0.53–1.52)	0.690	0.83 (0.47–1.46)	0.512
Motivation for distance teaching				
High	Reference			
Low	2.42 (1.32–4.42)	0.004*	2.42 (1.28–4.55)	0.006*
Moderate	1.36 (0.83–2.24)	0.217	1.31 (0.78–2.19)	0.300
No motivation	2.06 (0.86–4.93)	0.104	2.25 (088–5.78)	0.092

* Statistically significant *P*-value (*P* < 0.05).

**Table 3:** Psychological distress severity levels among the participants (*N* = 382) stratified by gender

Distress severity	Male (*N*)	Female (*N*)	Total (%)
No	66	50	116 (30.4)
Mild	46	33	79 (20.7)
Moderate	37	30	67 (17.5)
Severe	63	57	120 (31.4)
Total	212	170	382 (100)

### The emergency remote teaching strategy: Motivation and challenges.

University teachers were asked about the extent of their motivation for shifting the educational process into distance teaching (emergency remote teaching). The participant had to choose only one answer as follows: no, low, moderate, or high motivation. Interestingly, most participants responded with moderate to high motivation (*n* = 175, 45.8% and *n* = 103, 27.0%, respectively).

In addition, the multiple logistic regression analysis did not reveal any statistically significant association between the level of motivation for remote teaching and the examined predictors (Supplemental Material). Table 4 demonstrates a more descriptive analysis of the motivation for distance teaching stratified by distress severity and gender.

**Table 4:** Motivation for remote/distance teaching among the participants categorized by the psychological distress severity

Motivation	Distress severity				Total (%)
	No	Low	Moderate	Severe	
No	6	5	4	11	26 (6.8)
Low	17	13	15	33	78 (20.4)
Moderate	55	37	38	45	175 (45.8)
Strong/high	38	24	10	31	103 (27.0)
Total	116	79	67	120	382 (100)

Also, we asked the university teachers to report common challenges they faced or perceived while shifting from face to face to online distance teaching using a question with multiple choices in which the participant could have chosen all that applies. The majority of them (*n* = 317, 83.0%) were concerned about the increased possibility of cheating among students during online distance examinations, which is unfair for students. Moreover, they were challenged by the amount of time and effort needed to design examinations and fair assignments (*n* = 229, 59.9%). Also, 59.2% of participants perceived that this teaching strategy led to intrusion of their privacy; that is, students would contact the teachers at any time of the day irrespective to private personal time (e.g., rest time and family time) (see Table 5 for more details).

**Table 5:** Common challenges of remote/distance teaching as perceived by the participants

Item	Frequency (n)	Percentage
Online examinations increase the probability of cheating among students which is unfair	317	83.0
The need for more time and effort to design examinations and fair assessment tools	229	59.9
Intrusion of privacy	226	59.2
Reduced interaction with students	221	57.9
Ineffective communication with large numbers of students or colleagues	188	49.2
Anxiety about the quality of Internet service and video calls	169	44.2
Lack of technological competency and training to use e-learning platforms	46	12.0

Regarding the most used online platforms for delivering lectures or communicating with students, the academic staff could choose all that applies from multiple choices given. Interestingly, Moodle, Zoom, and Microsoft Teams were the most used e-learning platforms (*n* = 213, 55.8%; *n* = 198, 51.8%; *n* = 191, 50.0%, respectively) (see Table 6).

**Table 6:** Online platforms/software programs that were commonly used in remote/distance teaching and academic communication by the teachers

Program	Frequency (*n*)	Percentage
Moodle	213	55.8
Zoom	198	51.8
Microsoft Teams	191	50.0
Facebook/Facebook Messenger	164	42.9
WhatsApp Messenger	144	37.7
YouTube	53	13.9
Google Classrooms	34	8.9
Skype	16	4.2
Blackboard	12	3.1
Webex	6	1.6
Canvas	2	0.5
D2L (desire to learn)	1	0.3

### Self-adaptive activities and the perceived COVID-19–related concerns.

Regarding this topic, the university teachers were asked about 1) their coping activities during the COVID-19 quarantine and the suspension of on-campus educational activities at all HEIs, 2) whether or not they used medications to assist their coping strategies, and 3) their major source of distress or concerns during the pandemic. Concerning the coping activities, the respondents could choose all that applies from a list of choices. The most reported responses were more engagement with family (*n* = 240, 62.8%), using social media platforms (*n* = 216, 56.5%), talking to friends (*n* = 190, 49.7%), watching television (*n* = 172, 45.0%), prayers (*n* = 167, 43.7%), and home-based academic research activities (*n* = 133, 34.8%) (see Table 7).

**Table 7:** Self-adaptive activities of participants during the pandemic and its confinement measures

Activity	Frequency (*n*)	Percentage
More engagement with family	240	62.8
Using social media platforms	216	56.5
Talking to friends	190	49.7
Watching television	172	45.0
Prayers	167	43.7
Home-based academic research activities	133	34.8
Practicing sports at home	124	32.5
Reading books/novels	88	23.0
Listening to music	78	20.4
Herbal drinks	67	17.5
Increase smoking	49	12.8
Meditation	35	9.2
Practicing yoga	9	2.4
Talking to a psychological counsellor	1	0.3

**Figure 2.**
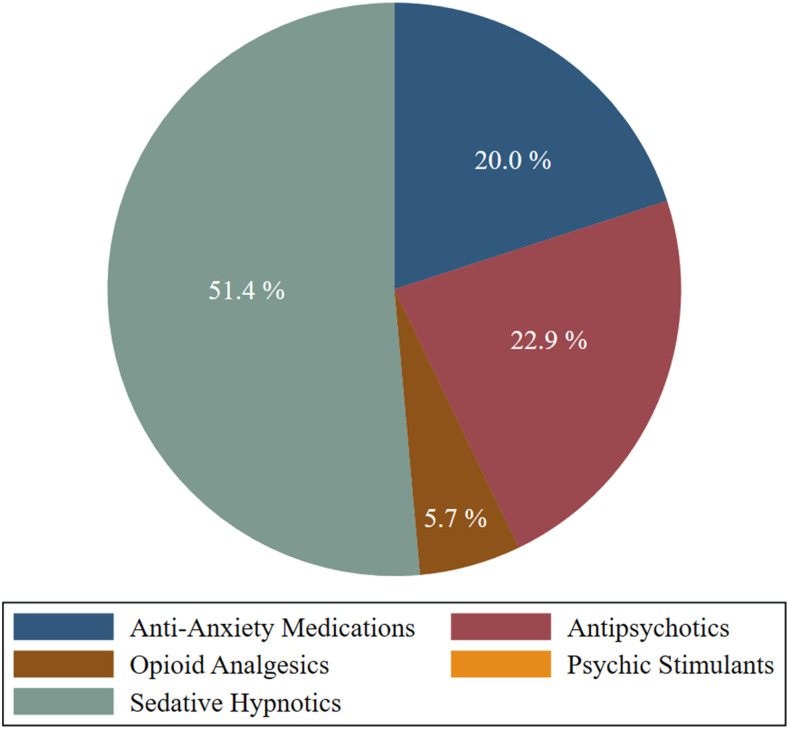
Types of medications used to support self-coping strategies among respondents (*n* = 38) due to COVID-19–induced distress.

Furthermore, among the 382 respondents, 344 participants (90.1%) reported no use of any medicinal drug in response to COVID-10–induced psychological distress, whereas 38 respondents (9.9%) reported using different medications, and sedative hypnotics (51.4%) were reported to be the most common type used—more details in Figure 2.

In response to a question about the major concerns as perceived by the academic staff, participants could choose all that apply from a list of choices as well as they were able to write any additional concerns not mentioned among choices; results showed that 215 (56.3%) reported infection by SARS-CoV-2 as their biggest concern, followed by 211 (55.2%) who reported social isolation (see Table 8).

**Table 8:** COVID-19–related concerns as perceived by the university teachers

Concern	Frequency (*n*)	Percentage
Infection by SARS-CoV-2	215	56.3
Curfew and social isolation	211	55.2
The economic impacts of COVID-19 pandemic	208	54.5
Inability to resume research activities	166	43.5
Distance teaching	130	34.0
Others (such as cancelled conferences and travel plans)	16	4.2

## DISCUSSION

Mental health conditions that are associated with outbreaks and pandemics have been recognized during previous pandemics in diverse vulnerable groups.^9,11,12,14,29,30^ Similarly, the association between the current COVID-19 pandemic with psychological distress, on different social components, including the general population, children, healthcare workers, older adults, patients, and students, is well established.^31–35^ Other published articles reported the impact of COVID-19 on the psychological status of individuals who already have a history of psychiatric disease.^13,29,31,36–38^ However, the psychological well-being of university teachers and the impacts of emergency remote education have not been thoroughly explored.

This study aimed to assess in what way the COVID-19 pandemic and the resultant quarantine/national measures would impact the psychological status of university teachers who are appointed at various HEIs in Jordan. Also, it aimed to assess their perceived motivation, challenges, and technostress due to distance teaching strategy along with their self-adaptive activities during this pandemic.

From March 2020 till early June 2020, Jordan declared a national defense law that enforced a total and then a gradual country lockdown, which included universities, schools, gyms, and shops.^4^ These unprecedented, extraordinary measures collectively have led to a significant leap in the society’s lifestyles, including academic life.

In our study, most of the participants (69.6%) faced various degrees of psychological distress amidst the current pandemic. As for coping mechanisms, most academics had more engagement with family as the main coping activity, followed by spending more time on social networking platforms, talking to friends, watching television, prayers, and home-based academic research activities. Around 9.9% of participants reported the use of medicinal drugs because of COVID-19–induced distress. The academic staff in this study showed a good attitude toward distance/remote teaching, as 72.8% had moderate to high motivation toward this educational strategy. However, there were some challenges and concerns that faced them. Namely, their worries about the increased likelihood of cheating among students during online examinations, the efforts and time needed to prepare online examinations and fair evaluation assignments, the intrusion of privacy, and reduced interaction with students. All these concerns were addressed in previous studies that assessed technostress related to online distance teaching.^15,39^

University teachers in our study expressed various pandemic-related concerns causing their distress. Interestingly, more than 55% reported infection by SARS-CoV-2, as one of their biggest concerns, followed by social isolation, and to a similar extent, the economic impact of the pandemic and curfew (e.g., reduced salaries, unsecured part-time teaching jobs, and a possible loss of research funds in some non–health-related academic departments), whereas around 43.5% of the academic staff were bothered by the inability to resume their research activities. Consequently, these concerns could lead to significant burden on the psychological status of the academic staff. In lieu of the limited literatures investigating the mental health impacts of COVID-19 on academic staff of HEIs, we considered some of the currently available published articles that addressed the psychological burden of the current pandemic on various social components.

Concerning the academic sector, recent literature by Cao et al.^14^ showed that anxiety was found in approximately 25.0% of college students in China during the current pandemic, as measured by the 7-item General Anxiety Disorder Scale. The anxiety level was correlated with different academic stressors. Similar to our study, there was no difference in the level of psychological distress between genders. Moreover, the level of anxiety was negatively associated with social support, which was also found to be one of the most common self-coping strategies reported by the university teachers in our study (engagement with family and social interaction). However, we found that moderate to severe distress affected around 48.9% of the academic staff and that the overall K10 score was negatively correlated with age. This higher distress proportion reported in our study (48.9%) than that in Cao et al.^14^ study (25.0%) is expected as academic staff might have more social as well as academic responsibilities and stressors than students. Besides, the scales used in the aforementioned studies were not the same, and the size of our sample was smaller than that in Cao et al.^14^ study. In addition, our survey was distributed during the final examination period, which might have led to a worsening in the teachers’ psychological status.

In addition, Schneider and Council^40^ have recently published a concise communication entitled *Distance learning in the era of COVID-19*, which demonstrated the impact of COVID-19 on education and negotiated some helpful tips in the transition to online learning. However, the implementation of distance learning and teaching strategy under COVID-19 control measures was not an easy mission. There were plenty of physical and psychological consequences related to this pandemic and its related containment/control measures. As revealed from the findings of our study, university teachers were not spared of such pandemic-related psychological distress during the implementation of strict mitigation measures, that is, cessation of on-campus academic activities. A study conducted by Hayes et al.^41^ found that the COVID-19 pandemic imposed more distress on individuals with insufficient experience for working from home before COVID-19 restrictions and who were females, which can be as a result of gendered roles at home in some cultural settings as well.

In the initial stages of the COVID-19 outbreak in China, Wang et al.^42^ conducted an online cross-sectional study that explored the degree of psychological impacts on public. Comparable to our findings, moderate to severe psychological distress was reported by 53.8% of respondents. Unlike our findings, female gender was correlated with higher degrees of distress at the beginning of the outbreak.

On the health workers’ perspective, psychological status was assessed in about 2,299 of the health workforce in China.^32^ Conceivably, medical staff had higher anxiety and depression than the administrative ones. Coping mechanisms or effects of certain stressors were not assessed in their study. Similar findings were reported among healthcare professionals in Singapore, in which 14.5% and 8.9% of participants had reported anxiety and depression, respectively.^33^ Still, the proportion of respondents with anxiety and depression in both of these studies was lower than those reported by our present study, and this could be due to the use of different assessment scales, the context where studies were performed as well as the different population studied.

To the best of our knowledge, this is the first original study to address the psychological status of university teachers (as a specific group) in Jordan in the midst of the current pandemic. In addition, it is the first to draw attention to the distress and challenges caused by the emergency remote teaching strategy at HEIs in Jordan. Yet, our study is not without limitations, which should be carefully considered. These include 1) using a non-randomized convenience sampling to recruit participants, which affects the representativeness of the sample as well as the generalizability of our results. However, we believed that it is the only sampling strategy attainable in light of the current situation of the pandemic, physical distancing, the suspension of face-to-face educational activities at all HEIs in the country, and moving toward online remote teaching platforms at the time of data collection 2) most of respondents were from universities/colleges located in the central governorates of Jordan; however, this indeed reflects the distribution of higher academic institutions (universities and colleges) in Jordan, with them being mostly congregated in the central region. 3) Using a cross-sectional study design which limits the ability of assessing temporality of events, 4) the survey represented self-reported states; thus, there might be underreporting of mental health well-being, and last, 5) we had a relatively small sample size which could be linked to the data collection period which occurred during final examinations (busy schedule of the academic staff), or the lack of interest among academic staff. Nevertheless, our study showed that the academic staff were prone to experience various levels of distress and disruptions in their psychological status during the COVID-19 pandemic, and they could be flagged as an underrepresented group. Also, our study findings open avenues for future large-scale research on this topic with a nationally representative sample.

Results from our study could help direct efforts of university management panel and decision-makers toward the psychological health issues in academic settings. Faculty members should also have more social support to assist them in overcoming the factor of social isolation, and this could be performed through weekly online social gatherings.

Psychological support services should be available to provide a proper help for the academic staff when needed. For instance, psychological support and counseling, as well as psychological health education, should be offered by universities and provided by specialists. Online training and workshops on how to manage and survive a distress during these extraordinary circumstances would be of a great benefit. Also, more efforts should be made to help the academics address their challenges regarding online remote teaching as well as aid them to brace technology and be more technologically competent, thus achieving equitable distance teaching and education for both the academic staff and the students.

Last, a more inclusive psychological support program on the national level should be developed and merged with other national efforts in mitigating COVID-19 and any future crisis in the country. As the lockdown and quarantine are being gradually relaxed in Jordan (and many other countries), thus, the psychological status is expected to be dynamic. So, it is intuitive to examine the effect of easing up the nationwide restrictions on HEIs (the current partial return to on-campus teaching, release of international travel ban, etc.) on the psychological well-being of the academic staff at HEIs in Jordan.

## CONCLUSION

The fear of COVID-19, the quarantine, and strict control measures, as well as social isolation that were forced during the current COVID-19 pandemic, could all result in the deterioration of the psychological status of various social components, including academicians. Our study highlighted various levels of psychological distress among the academic staff of HEIs in Jordan because of COVID-19.

Besides, the distance teaching strategy exhibited unexpected challenges and concerns among university teachers, as it was an unprecedented event in a country where most of the educational activities used to occur on campus. Most of university teachers in our study were highly concerned and fearful about SARS-CoV-2 infection, social isolation, as well as the economic impact of the pandemic; thus, prompt actions should be taken to promote their knowledge and awareness, increase distance social engagement, and securing part-time academic jobs. In addition, we recommend a nationwide mental health support plan to be part of the national response strategy and preparedness plan in combating any large-scale crisis in Jordan like the current pandemic, considering the general public, academic staff, students, healthcare workers, and other vulnerable groups in the community.

## Supplemental material

Supplemental materials

## References

[1] World Health Organization, 2020 Health Topics-Coronavrius Disease 2019. Geneva, Switzerland: WHO Available at: https://www.who.int/health-topics/coronavirus#tab=tab_1. Accessed June 17, 2020.

[2] Adhikari SP 2020 Epidemiology, causes, clinical manifestation and diagnosis, prevention and control of coronavirus disease (COVID-19) during the early outbreak period: a scoping review. Infect Dis Poverty 9: 29. 32183901 10.1186/s40249-020-00646-x PMC7079521

[3] Lai CC Shih TP Ko WC Tang HJ Hsueh PR, 2020 Severe acute respiratory syndrome coronavirus 2 (SARS-CoV-2) and coronavirus disease-2019 (COVID-19): the epidemic and the challenges. Int J Antimicrob Agents 55: 105924. 32081636 10.1016/j.ijantimicag.2020.105924 PMC7127800

[4] Al-Tammemi AB, 2020 The battle against COVID-19 in Jordan: an early overview of the Jordanian experience. Front Public Heal 8: 188. 10.3389/fpubh.2020.00188 PMC7220996 32574291

[5] World Health Organization, 2020 Coronavirus Disease (COVID-19) Outbreak Situation. Geneva, Switzerland: WHO Available at: https://www.who.int/emergencies/diseases/novel-coronavirus-2019. Accessed June 16, 2020.

[6] Kandel N Chungong S Omaar A Xing J, 2020 Health security capacities in the context of COVID-19 outbreak: an analysis of international health regulations annual report data from 182 countries. Lancet 395: 1047–1053. 32199075 10.1016/S0140-6736(20)30553-5 PMC7271261

[7] Prime Ministry of Jordan, 2020 Official Reports. Available at: http://www.pm.gov.jo/category/7603/اخبار.html. Accessed April 17, 2020.

[8] Jordanian Ministry of Health, 2020 COVID-19 in Jordan. Available at: https://corona.moh.gov.jo/ar. Accessed April 16, 2020.

[9] Zhang J Lu H Zeng H Zhang S Du Q Jiang T Du B, 2020 The differential psychological distress of populations affected by the COVID-19 pandemic. Brain Behav Immun 87: 49–50. 32304883 10.1016/j.bbi.2020.04.031 PMC7156946

[10] Fiorillo A Gorwood P, 2020 The consequences of the COVID-19 pandemic on mental health and implications for clinical practice. Eur Psychiatry 63: e32. 32234102 10.1192/j.eurpsy.2020.35 PMC7156565

[11] Ho CS Chee CY Ho RC, 2020 Mental health strategies to combat the psychological impact of COVID-19 beyond paranoia and panic. Ann Acad Med Singapore 49: 155–160. 32200399

[12] Ornell F Schuch JB Sordi AO Kessler FHP, 2020 “Pandemic fear” and COVID-19: mental health burden and strategies. Braz J Psychiatry 42: 232–235. 32267343 10.1590/1516-4446-2020-0008 PMC7236170

[13] Klomek AB, 2020 Suicide prevention during the COVID-19 outbreak. Lancet Psychiatry 7: 390. 10.1016/S2215-0366(20)30142-5 PMC7185940 32353271

[14] Cao W Fang Z Hou G Han M Xu X Dong J Zheng J, 2020 The psychological impact of the COVID-19 epidemic on college students in China. Psychiatry Res 287: 112934. 32229390 10.1016/j.psychres.2020.112934 PMC7102633

[15] Sahu P, 2020 Closure of universities due to coronavirus disease 2019 (COVID-19): impact on education and mental health of students and academic staff. Cureus 12: e7541. 32377489 10.7759/cureus.7541 PMC7198094

[16] Al-Jaghoub S Al-Yaseen H Hourani M El-Haddadeh R, 2009 E-learning adoption in higher education in Jordan: vision, reality and change. European and Mediterranean Conference on Information Systems (EMCIS) 2009, Izmir, Turkey. Available at: https://bura.brunel.ac.uk/bitstream/2438/4044/1/plugin-C83.pdf. Accessed September 13, 2020.

[17] Atoum A Al-Zoubi A Jaber MA Al-Dmour M Hammad B, 2017 A new approach for delivering eLearning courses in Jordanian universities. Adv Soc Sci Res J 4: 1–13.

[18] Ministry of Digital Economy and Entrepreneurship, 2017 ICT Households Survey. Available at: https://www.modee.gov.jo/content/studies-and-reports. Accessed March 25, 2020.

[19] Ministry of Higher Education and Scientific Research, 2018 Brief on Higher Education Sector in Jordan. Available at: http://www.mohe.gov.jo. Accessed March 21, 2020.

[20] Al Nawas B, 2020 Higher education council reviews distance learning experience. The Jordan Times. Available at: http://jordantimes.com/news/local/higher-education-council-reviews-distance-learning-experience. Accessed September 13, 2020.

[21] Chiappetta M, 2017 The technostress: definition, symptoms and risk prevention. Senses Sci 4: 358–361.

[22] Thornton L Batterham PJ Fassnacht DB Kay-Lambkin F Calear AL Hunt S, 2016 Recruiting for health, medical or psychosocial research using Facebook: systematic review. Internet Interv 4: 72–81. 30135792 10.1016/j.invent.2016.02.001 PMC6096238

[23] Ministry of Higher Education and Scientific Research, 2017 Education in Jordan. Available at: http://studyinjordan.jo/Default. Accessed September 13, 2020.

[24] National Comorbidity Survey, 2013 National Comorbidity Survey: Arabic K10. Available at: https://www.hcp.med.harvard.edu/ncs/k6_scales.php. Accessed May 2, 2020.

[25] Fassaert TJL de Wit MAS Tuinebreijer WC Wouters H Verhoeff AP Beekman ATF Dekker J, 2009 Psychometric Properties of an interviewer- administered version of the Kessler Psychological distress scale (K10) among Dutch, moroccan and Turkish respondents. Int J Methods Psychiatr Res 18: 159–168. 19701920 10.1002/mpr.288 PMC6878421

[26] Kessler RC Andrews G Colpe LJ Hiripi E Mroczek DK Normand SLT Walters EE Zaslavsky AM, 2002 Short screening scales to monitor population prevalences and trends in non-specific psychological distress. Psychol Med 32: 959–976. 12214795 10.1017/s0033291702006074

[27] Andrews G Slade T, 2001 Interpreting scores on the kessler psychological distress scale (K10). Aust N Z J Public Health 25: 494–497. 11824981 10.1111/j.1467-842x.2001.tb00310.x

[28] Easton SD Safadi NS Wang Y Hasson RG, 2017 The Kessler psychological distress scale: translation and validation of an Arabic version. Health Qual Life Outcomes 15: 215. 29078774 10.1186/s12955-017-0783-9 PMC5658946

[29] Brooks SK Webster RK Smith LE Woodland L Wessely S Greenberg N Rubin GJ, 2020 Rapid Review the psychological impact of quarantine and how to reduce it: rapid review of the evidence. Lancet 395: 912–920. 32112714 10.1016/S0140-6736(20)30460-8 PMC7158942

[30] Kim SW Su KP, 2020 Using psychoneuroimmunity against COVID-19. Brain Behav Immun 87: 4–5. 32234338 10.1016/j.bbi.2020.03.025 PMC7194899

[31] Rajkumar RP, 2020 COVID-19 and mental health: a review of the existing literature. Asian J Psychiatr 52: 102066. 32302935 10.1016/j.ajp.2020.102066 PMC7151415

[32] Lu W Wang H Lin Y Li L, 2020 Psychological status of medical workforce during the COVID-19 pandemic: a cross-sectional study. Psychiatry Res 288: 1–5. 10.1016/j.psychres.2020.112936 PMC7195354 32276196

[33] Tan BYQ 2020 Psychological impact of the COVID-19 pandemic on health care workers in Singapore. Ann Intern Med 173: 317–320. 32251513 10.7326/M20-1083 PMC7143149

[34] Qiu J Shen B Zhao M Wang Z Xie B Xu Y, 2020 A nationwide survey of psychological distress among Chinese people in the COVID-19 epidemic: implications and policy recommendations. Gen Psychiatry 33: e100213. 10.1136/gpsych-2020-100213 PMC7061893 32215365

[35] Lima CKT de Carvalho PM de Lima IAAS de Nunes JVAO Saraiva JS de Souza RI da Silva CGL Neto MLR, 2020 The emotional impact of coronavirus 2019-nCoV (new Coronavirus disease). Psychiatry Res 287: 112915. 32199182 10.1016/j.psychres.2020.112915 PMC7195292

[36] D’Agostino A Demartini B Cavallotti S Gambini O, 2020 Mental health services in Italy during the COVID-19 outbreak. Lancet Psychiatry 7: 385–387. 32353266 10.1016/S2215-0366(20)30133-4 PMC7185925

[37] Yao H Chen JH Xu YF, 2020 Patients with mental health disorders in the COVID-19 epidemic. Lancet Psychiatry 7: e21. 32199510 10.1016/S2215-0366(20)30090-0 PMC7269717

[38] Zhu Y Chen L Ji H Xi M Fang Y Li Y, 2020 The risk and prevention of novel coronavirus pneumonia infections among inpatients in psychiatric hospitals. Neurosci Bull 36: 299–302. 32096116 10.1007/s12264-020-00476-9 PMC7056754

[39] Dragano N Lunau T, 2020 Technostress at work and mental health: concepts and research results. Curr Opin Psychiatry 33: 407–413. 32324623 10.1097/YCO.0000000000000613

[40] Schneider SL Council ML, 2020 Distance learning in the era of COVID-19. Arch Dermatol Res 1–2 (Epub ahead of print). 10.1007/s00403-020-02088-9. 32385691 PMC7209972

[41] Hayes S Priestley JL Ishmakhametov N Ray HE, 2020 “I’m not working from home, I’m living at work”: perceived stress and work-related burnout before and during COVID-19. PsyArXiv (Preprint). 10.31234/osf.io/vnkwa.

[42] Wang C Pan R Wan X Tan Y Xu L Ho CS Ho RC, 2020 Immediate psychological responses and associated factors during the initial stage of the 2019 coronavirus disease (COVID-19) epidemic among the general population in China. Int J Environ Res Public Health 17: 1729. 10.3390/ijerph17051729 PMC7084952 32155789

