# Supplementary material for: The Impact of the COVID-19 Pandemic and Emergency Distance Teaching on the Psychological Status of University Teachers: A Cross-Sectional Study in Jordan
Source: Am J Trop Med Hyg. 2020 Oct 27;103(6):2391–9. doi: 10.4269/ajtmh.20-0877 (PMC7695050; doi:10.4269/ajtmh.20-0877)
Supplement: Supplementary file 1 [file tpmd200877.SD1.pdf]

Supplementary Material

Supplementary Table. Results of Multiple Logistic Regression for the association between Motivation for Distance Teaching (recoded as binary outcome variable) and multiple predictor variables

Predictors	Crude OR (95% CI)	P-value	Adjusted OR (95% CI)	P-value
**Age**				
25-34	**Reference**			
35-44	0.81 (0.42-1.55)	0.523	0.61 (0.29-1.26)	0.181
45-54	1.39 (0.67-2.89)	0.371	0.98 (0.41-2.32)	0.957
55-64	1.17 (0.50-2.75)	0.718	0.87 (0.28-2.71)	0.808
65-75	0.44 (0.12-1.64)	0.224	0.40 (0.07-2.26)	0.298
**Gender**				
Female	**Reference**			
Male	0.88 (0.56-1.39)	0.598	0.72 (0.43-1.20)	0.206
**Academic Rank**				
Assistant.Prof	**Reference**			
Associate.Prof	1.09 (0.59-2.01)	0.788	0.96 (0.50-1.85)	0.907
Full.Prof	0.98 (0.53-1.84)	0.959	0.87 (0.38-1.99)	0.745
Lecturer (MSc)	0.67 (0.36-1.24)	0.207	0.52 (0.26-1.03)	0.062
**Scientific Discipline**				
Humanities	**Reference**			
Medical	0.80 (0.47-1.38)	0.423	0.78 (0.44-1.38)	0.388
Sciences	1.05 (0.57-1.91)	0.878	1.15 (0.61-2.16)	0.660
**Duration of Experience**				
1-15 years	**Reference**			
16-35 years	1.56 (0.85-2.85)	0.154	1.44 (0.65-3.17)	0.370
36-53 years	0.66 (0.28-1.57)	0.344	0.73 (0.21-2.54)	0.623
